# Changes in Physical Activity and the Occurrence of Specific Symptoms of “Long-COVID Syndrome” in Men Aged 18–25

**DOI:** 10.3390/ijerph19031199

**Published:** 2022-01-21

**Authors:** Anna Sojka, Mariusz Machniak, Waldemar Andrzejewski, Aureliusz Kosendiak, Agnieszka Chwałczyńska

**Affiliations:** 1 Scientific Circle at the Department of Human Biology, Faculty of Physiotherapy, Wroclaw University of Health and Sport Sciences, 51612 Wrocław, Poland; anna.sojka98@gmail.com (A.S.); machniak.mariusz@gmail.com (M.M.); 2 Faculty of Physiotherapy, Wroclaw University of Health and Sport Sciences, 51612 Wrocław, Poland; waldemar.andrzejewski@awf.wroc.pl; 3 Department of Physical Education and Sport, Wroclaw Medical University, 51601 Wrocław, Poland; aureliusz.kosendiak@umed.wroc.pl

**Keywords:** COVID-19 restrictions, physical activity after the pandemic, long-COVID syndrome

## Abstract

The aim of this study was to assess the occurrence of non-specific symptoms of “long-COVID syndrome” depending on the physical activity undertaken resulting from the imposed forms of study (distance learning–contact learning); 136 men aged 21.5 ± 1.58 from universities educating students of medical faculties were examined. The difference between the universities was mainly due to the nature of the classes undertaken (classes remotely-hybrid form) in the period from March 2020 to February 2021. Among the respondents, 17% in Group I and 16% in Group II were infected with the SARS-CoV-2 virus, including 50% in Group I with moderate symptoms, and in Group II—most people 45% with mild symptoms. The conducted research clearly shows the impact of the COVID-19 pandemic on students. They show a number of important problems, such as reduced physical activity, as well as increased body weight and time spent in front of the monitor. They also make it clear that the health consequences of the pandemic affect both people who were infected with the SARS-CoV-2 virus and those who did not suffer from this infection.

## 1. Introduction

At the beginning of 2020, the health situation in the world changed significantly and the appearance of the SARS-CoV-2 virus affected the daily functioning of people [1,2]. The rapid spread of the virus led to the introduction of a pandemic state by the World Health Organization (WHO) in March 2020 [3]. On 11 March 2020, Poland introduced restrictions related to the COVID-19 pandemic caused by the SARS-CoV-2 virus [4,5]. The need for isolation initiated by the COVID-19 pandemic was associated with the introduction of many changes that determined everyday functioning in the new reality. One of the WHO recommendations to prevent transmission of the coronavirus was to stay at home and only leave when necessary, which in turn involved limiting physical activity (PA) during the day [6]. Given the continued spread of the coronavirus, it was an important aspect in preventing progressive infections; however, it is known that regular PA is an equally important preventive strategy for maintaining a satisfactory level of not only physical but also mental health [7]. The WHO recommends that, to maintain good health, people over the age of 18 should regularly engage in PA and devote at least 150 to 300 min a week of moderate-intensity aerobic exercise for significant health benefits. At the same time, in the latest WHO recommendations, attention is paid to exercises to strengthen the muscles of all main groups and limiting the amount of time spent in a sitting position. According to the recommendations, the alternative to hours spent in a sitting position is to replace them with any physical activity, even low-intensity, or to introduce an equivalent activity of moderate to high intensity in an amount much greater than the recommendations [3]. Regular PA improves health-related quality of life because it induces physiological cardioprotective responses, such as increased stroke volume and output, and decreased heart rate and blood pressure [8]. Research shows that the introduction of quarantine significantly reduced the total weekly energy expenditure on PA in all age groups, especially in men. People participating in the study also experienced a decrease in well-being [7,9,10], and the amount of time spent sitting also increased significantly. Subsequent studies show that extending the period of staying at home leads to a reduction in PA and an increase in the level of stress, which consequently increases the risk of chronic diseases and, consequently, deteriorates the quality of life [8,11,12,13]. Yet another study indicates that a reduction in the amount of PA is especially felt by people who are active on a daily basis, with a decrease in sleep quality and a decrease in overall self-esteem [14,15,16]. Deterioration of well-being and sleep quality are also among the many symptoms of long-COVID syndrome. According to the latest literature, it is a syndrome with a diverse set of symptoms lasting more than 12 weeks after the diagnosis of COVID-19 infection [17,18,19,20]; however, in some patients, it happens that the signs of long-COVID syndrome do not appear after 12 weeks, but only 3–4 weeks later. Common symptoms in people with long-COVID syndrome are profound fatigue, shortness of breath, cough, chest pain, palpitations, headache, joint pain, muscle aches and weakness, insomnia, tingling, diarrhea, rash or hair loss, imbalance and gait, neurocognitive problems, including problems with memory and concentration, and a deterioration in quality of life. People with long-COVID syndrome may have one or more of the symptoms listed, and the sheer multitude of them shows how complex the problem is. Scientists have distinguished two main patterns of symptoms of long-COVID syndrome. The first is fatigue, headache, and upper respiratory problems including shortness of breath, sore throat, persistent cough, and loss of sense of smell. In contrast, the second pattern is multi-system ailments, including persistent fever, as well as gastroenterological symptoms [21,22].

The aim of this study was to assess the occurrence of non-specific symptoms of “long-COVID syndrome” depending on the PA undertaken resulting from the imposed forms of study (distance learning–contact learning).

## 2. Materials and Methods

**Figure 1:**
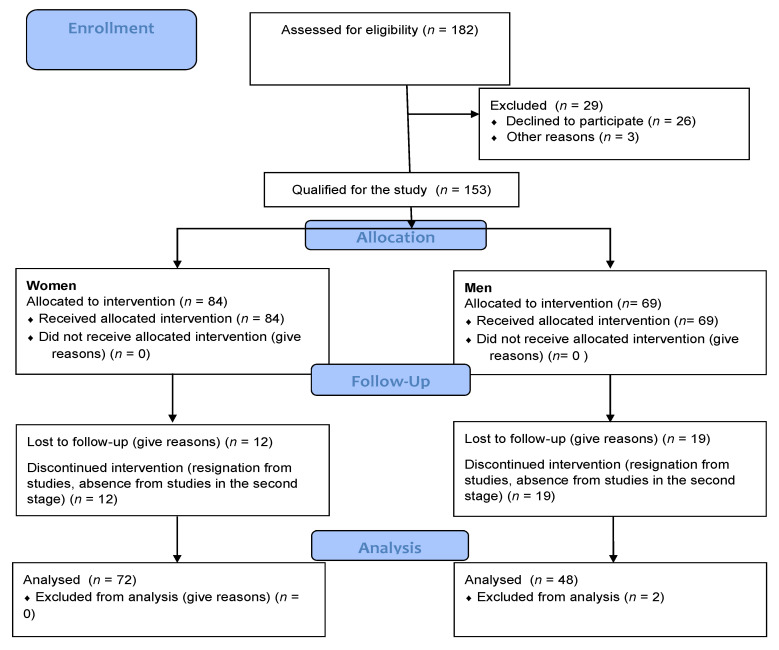
Consor diagram.

The 136 men aged 21.5 ± 1.58 from universities educating students of medical faculties were examined. The research was carried out at the end of February and at the beginning of March 2020 (ended before 11 March 2020, i.e., before the introduction of the remote teaching authority) and in March 2021, i.e., right after loosening the restrictions giving the possibility of conducting hybrid classes for medical faculties at universities other than universities medical. All respondents, in the first stage of the study, were first-year students and had an occupational medicine medical certificate confirming the ability to undertake studies. Before the start of the study (in 2020 and 2021), each respondent signed a consent to the study with a statement that there are no contraindications to participate in the study and to take PA. The research was carried out during the classes carried out during the studies, none of the project participants showed signs of disease (increased temperature, runny nose, cough, diagnosed chronic disease). In the case of the study in 2021, additionally, at the entrance to the classes, during which the study was conducted, a control measurement of body temperature was performed—among the respondents, the temperature was not higher than 36.8 °C. None of the respondents reported deterioration of health—the presence of chronic diseases. At the same time, in 2021, only students without symptoms were allowed to attend classes. People who had contraindications to practice PA were excluded at the recruitment stage (Figure 1). In the event of a chronic disease, the subjects most often discontinued their studies, which was also shown in the CONSOR diagram (Figure 1). The selection of the group of medical students was dictated by their knowledge of the importance of PA in the quality of life. The respondents were divided into two groups depending on the university. Group I (*n* = 70–51%)—students of the Faculty of Physiotherapy, Wroclaw University of Health and Sport Sciences, Group II (*n* = 66–49%)—students at the Wroclaw Medical University. The division was dictated by the nature of the home university: Wroclaw University of Health and Sport Sciences is a sports university and Wroclaw Medical University—typically medical, but both of these universities educate students in medical faculties (physiotherapy, medicine). The difference between the universities was mainly due to the nature of the classes undertaken in the period from March 2020 to February 2021. During this period, the Wroclaw University of Health and Sport Sciences conducted classes remotely, regardless of the type and nature of the subject (no classes in contact with the patient), while the Wroclaw Medical University conducted classes in a hybrid form—remote lectures and classes with patients in contact with the sanitary regime.

The respondents completed a questionnaire consisting of 24 questions on PA (author’s questionnaire based on IPAQ) [23], lifestyle, and changes they observed during the lockdown period caused by the COVID-19 pandemic. The survey covered three periods related to the restrictions introduced in the country: period I—before the occurrence of diseases caused by SARS-CoV-2, to March 2020, which marked the introduction of the first restrictions suspension of education at universities; period II—the complete suspension of classes and closure of sports centers (March 2020–February 2021); period III—after returning to science in a hybrid form and when the restrictions are relaxed, the opening of outdoor sports centers (February–April 2021). In the survey, apart from the information about undertaking physical activity, the number of hours spent in front of the monitor, the question was asked about the presence of non-specific symptoms appearing in the diagnosis of post-COVID syndrome. The symptoms of this disease were classified as previously unprecedented symptoms from the following side: respiratory system (cough, chronic fatigue, shortness of breath), circulatory system (chest pain, palpitations), nervous system (syncope, headache, dizziness, temporary memory loss, cognitive impairment, great problems with concentration, depressed mood, apathy, irritability, loss of sense of smell or taste), the musculoskeletal system (muscle weakness, muscle pain, numbness in the limbs, tingling extremities, joint pain, joint swelling), digestive system (significant loss of appetite, abdominal pain, diarrhea, vomiting, sore throat) and skin and mucus symptoms (skin rashes, skin changes on the arms/legs, face swelling, dry, red lips) or depression [24,25,26,27]. In the survey, the respondents determined their body height and weight in March 2020 and March 2021, the values provided by the respondents were verified on the basis of weight and body composition using an 8-electrode body composition analyzer—MC-780 by TANITA and SECA 213 stadiometer tests carried out in the same periods on the majority of respondents The study was conducted in accordance with the Declaration of Helsinki, and the research project received approval from the Senate’s Bioethical Committee at the Academy of Physical Education in Wroclaw (no.12/2019; date of ethical approval 15.03.2019). Signed informed consent forms were obtained from all participants. The study was conducted in accordance with the CONSORT (Consolidated Standards of Reporting Trials) statement (Figure 1).

### Statistical Methods

The results were prepared with the use of the Statistica 13.3 package. The frequency tables and descriptive statistics (mean ± SD) were used to describe the group. Non-parametric tests for dependent groups were used to compare different periods. Statistical significance was determined using the chi-Pearson square test, student’s *t*-test and Friedman ANOVA.

## 3. Results

Among the respondents, 17% in Group I and 16% in Group II were infected with the SARS-CoV-2 virus, including 50% in Group I with moderate symptoms, and in Group II—45% with mild symptoms. The onset of the disease was 4 ± 3.08 months before the second series of tests. None of the subjects required hospitalization, and the mean duration of symptoms was 10 days.

Within the last 6 months, 50% of the respondents noticed non-specific symptoms of post-COVID syndrome from at least two systems. In group I, the most common were headaches (31.4%) and symptoms related to the locomotor system—muscle weakness, tingling in the limbs, muscle pain, general fatigue. In group II, headaches occurred in 16.7% of respondents, abdominal pain and symptoms from the nervous system were also frequent, including chronic fatigue, dizziness, numbness in the limbs. Statistically significant differences were observed in the number of people reporting headaches (*p* = 0.045), muscle pain (*p* = 0.027) and muscle weakness (*p* = 0.046). The results are presented in Table 1. The remaining symptoms appeared less frequently than in 10% of the subjects in each group.

An important element is the appearance of depressive symptoms in 15.7% of respondents in Group I and 10.6% in Group II.

Taking into account the conscious illness of SARS-CoV-2 virus infection, it can be noticed that 70% of diagnosed men developed non-specific symptoms of long-COVID syndrome, i.e., damage from at least two systems, and 40% developed 5–10 symptoms. The occurrence of the most common symptoms of long-COVID syndrome is presented in Table 1.

In the studies preceding the SARS-CoV-2 virus pandemic, lower body weight was recorded in Group I, and after a period of limited activity and hybrid teaching, subjects in Group II had lower body weight, but these differences were not statistically significant. A statistical significance was observed in the change of body weight over the study period in Group I. Body weight during the lockdown period increased in 51% of subjects from Group I by an average of 2.97 ± 2.4 kg and 45.4% of men from Group II by an average of 2.68 ± 1.75 kg. Every fifth subject from Group I and every third subject from Group II decreased their body weight. The mean differences in body weight between the groups are statistically significantly different. Body weight and BMI results are presented in Table 2.

An important element in assessing the impact of restrictions resulting from the pandemic on the lifestyle of young people was the change in sports and social activity of the respondents. The research shows that, before the pandemic period, the subjects from Group I statistically significantly spent more time on PA and meeting friends outside the place of residence, regardless of the day of the week, compared to Group II. At the same time, in Group I, the greatest changes in the time spent leaving home and physical activity, depending on the pandemic situation, were observed.

In the pre-pandemic period, the subjects in both groups performed PA at the frequency, intensity, and time recommended by the WHO (at least 150 to 300 min a week in 3 times/per week sessions). It should be noted, however, that the respondents from Group I devoted an average of 240 min/per week to physical activity, while in Group II the average of 168 min/per week was devoted to physical activity, which is a statistically significant difference. With the introduction of restrictions related to the pandemic, the subjects in Group I statistically significantly reduced (by an average of 100 min/per week) the amount of time spent on physical activity, while in the latter group the change was insignificant. The results are presented in Table 3.

The introduced restrictions also affected the time spent away from home by the respondents from Group I. The average time spent away from home decreased by half; however, these changes are not statistically significant as the values are too varied. No significant changes were observed in Group II. The results are presented in Table 3.

Statistically significant changes were also observed in the Screen time. In the case of Group I, the change occurred as a result of the introduction of remote learning and the transfer of all classes in the period March 2020–February 2021 to educational platforms. Once again, the reduction in the number of minutes spent in front of the monitor was observed when the activities in the hybrid form were introduced lectures continued as webinars and practical classes in contact with the patient. In the case of Group II, the time in front of the monitor changed slightly but significantly. The results are presented in Table 3.

There was no correlation between the occurrence of symptoms and the time between onset and examination. There was no correlation between the incidence and number of symptoms and body weight and BMI.

A weak statistically significant correlation was observed between the number of symptoms and the length of Screen time during the week (0.291) and at the weekend (0.287). There were weak statistically significant correlations between the increase in body weight (0.208) and the increase in BMI (0.214) and the number of hours spent in front of the computer during the week.

## 4. Discussion

In order to minimize the spread of COVID-19 disease caused by the SARS-CoV-2 virus, the WHO has recommended social distancing and leaving home only when necessary [6]. Researchers from Spain emphasize that, especially in young adults, this was associated with a number of changes in everyday activities, which led to reduced physical activity, more frequent sitting, and increased use of screen devices [28].

This was also the case with the respondents from Group I, who in the period March 2020–February 2021 attended the classes remotely. Then, the teaching mode was changed to a hybrid one, which assumed that classes were conducted in contact with patients in accordance with the sanitary regime, and the lectures continued online. On the other hand, for the subjects from Group II, the classes were conducted in a hybrid manner from the very beginning. The introduction of distance learning has limited the daily PA. Students from group I did not have to move to the city where the University was located, they stayed in their family homes, while ministerial orders limited interpersonal contacts. As a result, the respondents moved their activity into virtual space, increasing the time spent in front of the computer.

Among the respondents in Group I who suffered from the coronavirus, 50% reported the presence of moderate symptoms of the disease, while 45% in Group II reported only a slight worsening of its symptoms.

Among 50% of the respondents, including those who did not suffer from COVID-19, non-specific symptoms of post-COVID syndrome were observed from at least two systems. The most frequently reported symptom was headache, and in Group I also complaints from the musculoskeletal system, while in Group II it was additionally abdominal pain and ailments from the nervous system. Statistically significant differences concerned the number of people reporting headaches, muscle pain and muscle weakness. Similar studies were carried out by Chwałczyńska and Andrzejewski on a group of women and men participating in distance learning at a sports and medical university. In their research, the authors observed a variety of symptoms depending on the sex of the subjects. In women, symptoms from the nervous system were more common—headaches and dizziness or chronic fatigue, and in men—symptoms from the musculoskeletal system—pain in muscles, joints, numbness of the limbs or chronic fatigue. It can also be observed that the results obtained by Chwałczyńska and Andrzejewski in the group of men coincide with the results presented in this study of the respondents from Group I, and the results of women with the data obtained in Group II [1]. Such a difference between the results in the group of men may be caused by the nature of the university where the respondents study. Group I are medical students, but at a sports university, group II is a medical university. Although there are the same faculties at both universities, men with a higher PA choose a sports university, and those who do not do sports chose a medical university. Therefore, the introduced restrictions, and in particular the closure of sports centers, had a negative impact on the amount of time spent by the subjects from group I on PA. The decrease in PA in this group could have contributed to the appearance of symptoms related to the locomotor system. The organism adapted to daily PA, deprived not only of sports activities, but also of daily movement, shows symptoms of malfunctioning in the locomotor system, and the same reaction was observed in men in group I. “long-COVID syndrome” and PA change. However, the appearance of ailments from the skeletal system in group I may not be a post-COVID symptom, but rather result from the limitation of physical effort. Non-specific symptoms of “long-COVID syndrome” depend, just like the symptoms of Sars-Cov-2 infection, on the condition of our body. This virus attacks the weakest, most susceptible to infection organs of the body, it may also increase the sensitivity of the locomotor system to infection, and thus the occurrence of post-COVID symptoms. It is worth noting here that in the study, the group of people with the symptoms of long-COVID syndrome was classified in men with non-specific symptoms from at least two systems at the same time. Therefore, it is possible to associate the appearance of skeletal abnormalities with the incidence of Sars-Cov-2 infection.

The research on the impact of the introduced restrictions on physical health should be extended, because reports on the negative effects of the restrictions on mental health, the level of perceived stress, and sleep quality constitute a numerical advantage [1]. These issues are discussed more extensively by Casagrande et al., where 57.1% of study participants reported poor sleep quality, 32.1% of people experienced high anxiety, and 41.8% had high levels of stress [29]. Similar studies on the impact of restrictions on the mental state were presented in the work of Jiao et al., noting the appearance of sleep disorders, increased irritability or anxiety in children [30].

Non-specific symptoms of long-COVID syndrome, from at least two systems, were reported by 70% of all male respondents who contracted the SARS-CoV-2 virus. Moreover, in 40% of respondents, it was from 5 to 10 symptoms. Similar results were also obtained by Carfi et al., where 32% of respondents had 1 or 2 symptoms of post-COVID syndrome, and 55% had 3 or more symptoms [23].

Among the subjects, changes in body weight were also determined in the period from March 2020 to April 2021. Before the pandemic period, lower weight was recorded in subjects in Group I, while after the period of remote learning it exceeded the body weight of subjects in Group II; however, these differences were not statistically significant. The differences in the body weight of the surveyed men between the groups are most likely caused by regularly practiced PA among the respondents from the sports university. On the other hand, the changes in body weight in Group I over the studied period turned out to be statistically significant, and during the lockdown it increased by an average of 2.97 ± 2.4 kg in 51% of respondents. The change in body weight in this group was most likely caused by the change in the form of PA in the subjects. The closure of sports centers such as gyms, gyms and swimming pools forced a change from group activities to individual activities such as running, walking, outdoor gyms in good weather, cycling or home exercises. Such forms, implemented individually, often have a lower intensity, which can be observed in the increased body weight of the subjects. In the subjects in Group II, it was on average 2.68 ± 1.75 kg and it concerned 45.4% of men. The mean differences in body weight in the period from March 2020 to April 2021, both in groups I and II, also turned out to be statistically significant. Studies on weight change during a pandemic were also carried out by He et al., who showed that men with a BMI <24 gained weight [31]. Other studies by Deschasaux-Tanguy et al. indicate that 35% of participants increased their body weight and also observed increased snacking, decreased consumption of fresh food, and increased consumption of sweets. However, the same research shows that 23% of people saw a decrease in their body weight [32]. Such people were also found among the respondents from groups I and II, because the study showed a decrease in body weight in every 5 of the subjects in Group I and every 3 in Group II. The change in body weight is primarily a consequence of limiting physical activity, i.e., a change in lifestyle that was forced by the restrictions caused by the COVID-19 pandemic. At the same time, people who have been infected with the SARS-CoV-2 virus, especially with respiratory symptoms, may have gained weight secondary to a reduction in exercise capacity.

As a result of the introduced restrictions, aimed at minimizing the spread of the SARS-CoV-2 virus, the respondents in Group I significantly reduced the amount of time spent leaving the house and physical activity. In the period before the pandemic, the amount of this time was statistically significantly greater than in the case of respondents from Group II. PA of students during the pandemic was also investigated by Lopez-Valenciano et al., who showed that 9 out of 10 studies showed a significant reduction in physical activity. Compared to the pre-lockdown period, 5 studies showed a reduction in light physical activity, while as many as 7 studies showed a reduction in high PA [33]. Restricting the ability to leave the house may have contributed to the appearance of depressive symptoms. This was observed in their work by Chwałczyńska and Andrzejewski, especially in the group of women studied, where the percentage of people with depressive symptoms was higher than in previous studies on similar groups [1]. At the same time, people who contracted the infection reported chronic fatigue, which could have a negative impact on the quality of life and lead to depression. It should also be remembered that taking PA has a positive effect on the quality of life and thus may reduce the risk of depression. Many authors emphasize that the stress caused by the uncertainty we encountered during the pandemic could not only affect the emergence of depressive states, but also the reduction of immunity, and thus greater susceptibility to complications after infection [2,7,10,14,16,29,30]. The restriction on taking up PA introduced by the government as well as the introduction of distance learning resulted in a stressful situation, which could have a negative effect on the immune system of the respondents and the occurrence of post-COVID syndrome symptoms.

Before the pandemic, the subjects from Group I devoted statistically significantly more time to PA and it was on average 240 min/per week, where in Group II this time was on average 168 min/per week. The introduction of restrictions did not statistically significantly change the time devoted to PA in Group II, which could have been caused by the contact behavior of the classes. The lack of a statistically significant change in the time spent on PA was also caused by a significantly lower activity before the introduction of restrictions. The respondents from this group, apart from limiting the possibility of meeting in restaurants or bars, did not change their activity. Before the pandemic, most of them only realized sports activities as part of their studies, and this was only limited to them because sports activities were carried out remotely. On the other hand, in Group I, where full remote learning with the use of an educational platform was introduced, the time spent on PA was shorter on average by 100 min/per week. The average time spent outside the home was halved, but these changes are not statistically significant. Changes in PA were also checked by Lesser et al., who divided the subjects into active and physically inactive people, according to the level of PA undertaken before the pandemic. The research shows that among inactive people, 40.5% of participants became even more inactive, while 33% increased their physical activity. In turn, in the group of active people, 40% of people increased their activity and decreased by 22.4%. Moreover, despite the introduced restrictions, 39.6% of people in the active group remained with their typical choice of PA [34]. Similar results were obtained by Chwałczyńska and Andrzejewski in a study conducted on a group of students of a sports and medical university, where the respondents changed their forms of activity and the time devoted to its implementation remained unchanged [1].

As a result of the introduction of distance learning, the amount of Screen time has also statistically increased. These values only decreased after changing the class mode to hybrid. In the subjects in Group II, the Screen time also changed significantly, although less than in the case of students from a sports university. The increase in the Screen time was noted by researchers dealing with children [35,36,37,38,39]. In their research, Qin et al., from the initial period of the pandemic in China, noted a significant increase in the Screen time, especially in the group of young adults, with a simultaneous decrease in physical activity, which translated into an emotional state, the occurrence of depression and even loss of health [40]. Research conducted by SteamDB platforms has observed a significant increase in active participants in online games during the pandemic. This may prove not only the increased interest in e-sport, but most of all the search for interpersonal contacts through safe, non-contact connections. According to researchers, turning to online games is also a way to diversify the time spent at home during a forced lockdown [41]. Increasing the time spent in front of the computer may have contributed to the appearance of the symptoms of post-COVID syndrome. Forced sitting in front of the computer had a negative impact on body weight, as well as on physical performance and lung capacity.

In the conducted studies, no correlation was found between the occurrence of symptoms and the time between onset and examination, as well as between the occurrence and number of symptoms and body weight and BMI. On the other hand, a weak but statistically significant correlation was observed between the number of symptoms and the length of Screen time during the week and at the weekend. We also found weakly statistically significant correlations between the increase in body weight and the increase in BMI, and the number of hours spent in front of the monitor during the week. An additional, but significant result obtained from the research is the occurrence of depressive symptoms, which were reported by 15.7% of the respondents in Group I and 10.6% in Group II. Disturbing reports on the impact of restrictions on mental health can also be found in studies conducted by Sun et al., where 46.55% of students from 19 different universities in China reported depressive symptoms, and in addition 67.05% of people experienced traumatic stress, 24.73% struggled with anxiety symptoms and 19.56% of the students had suicidal thoughts [42].

An aspect that requires more detailed research is the changes in the amount of time spent outside the home due to the restrictions introduced, but a larger number of variables should be analyzed to avoid large variations in values. 

The study was conducted on a selected group of medical students, i.e., individuals with knowledge about health aspects, the importance of PA and its role in the quality of life and maintaining good health, which could have influenced the results obtained. However, the selection of the group was dictated by the form of training during the pandemic period. Only medical universities (group II) conducted classes in the form of student-teacher contact, while group I consists of medical students who conducted their studies remotely. The part common to both groups was the medical field of study the difference was the form of study during the pandemic period. Although Group II had standard classes, it did not have contact with the patient, because they were first-year students. Their classes were held in contact, but only with the lecturer, they were, among others, classes in an anatomical laboratory, first aid classes on mannequins, histology, history of medicine or genetics, which did not require contact with the patient. Group I are people who, like the rest of the country, were subjected to governmental persons aimed at limiting interpersonal contacts due to the pandemic.

In order to generalize the results, people of comparable age studying non-medical subjects and non-students working would also need to be surveyed.

One of the limitations of the described studies, conducted by a research team consisting of physiotherapists, was the lack of an objective assessment of the health of the subjects. With the consent of the bioethics committee, the subjective assessment of the health was limited as the subjects were adults and consciously signed the health declaration—no contraindications to participate in the research project. In the future, the research team should be expanded to include a physician assessing the current state of health of the respondents. However, the research project started before the SARS-CoV-2 pandemic and was modified due to the pandemic situation.

The main problem with extending the research is the lack of preliminary research done before the pandemic. The research from 2020 used in this publication was part of a larger research project: the assessment of the segmental body composition, using the FFF index, depending on the physical activity undertaken. Pre-pandemic testing cannot be rerun as it is impossible to go back to the pre-pandemic state

## 5. Conclusions

The conducted research clearly shows the impact of the COVID-19 pandemic on students. They show a number of important problems, such as reduced physical activity, as well as increased body weight and Screen time. They also make it clear that the health consequences of the pandemic affect both people who were infected with the SARS-CoV-2 virus and those who did not suffer from this infection.

## Figures and Tables

**Table 1:** The most common non-specific symptoms of “long-COVID” in the studied men.

	Division According to the University			Classification Due to the Diagnosis of SARS-Cov-2 Virus Infection		
	Group I (%)	Group II (%)	*p*	Diagnosed, Contracted COVID-19 (%)	Undiagnosed (%)	*p*
chronic fatigue	24.3	13.6	NS	40	15.6	0.01
breathlessness				10	1.7	0.04
pain in the chest				15	3.4	0.03
headaches	31.4	16.7	0.045	30	23.3	NS
dizziness	12.8	13.4	NS	20	12.1	0.003
depression	15.7	10.6	NS	15	12.9	
muscle weakness	21.4	9.1	0.046	25	13.8	
muscle aches	18.6	6.1	0.027	30	9.5	0.01
numbness in the limbs	10	3	NS	5	6.9	NS
tingling in the limbs	21.4	12.1	NS	25	15.5	NS
joint swelling				15	0.0	0.000
stomach pain	20	12.1	NS			
taste disturbance				20	2.6	0.001
smell disorders				20	3.4	0.003
dry cough	10	10.1	NS	20	8.6	NS
sore throat	17.1	10.1	NS	20	12.9	NS

**Table 2:** Body weight and BMI in the studied men before and after the lockdown period.

	Group I		Group II		*p*
	Average ± SD	Min-Max	Average ± SD	Min-Max	
Body weight before (March 2020) (kg)	77.48 ± 8.76	52.5–99.9	78.51 ± 13.43	53.0–116.0	0.636
Body weight (April 2021) (kg)	80.85 ± 10.47	51.0–104.8	77.90 ± 12.79	53.0–114.0	0.147
	0.040 *		0.272		
Body weight difference (2020–2021)	1.15 ± 3.84	8.7 + 10.1	0.61 ± 4.48	−20.0 + 7.0	0.028 ^#^
BMI (March 2020)	23.39 ± 2.48		23.78 ± 3.76		0.528
BMI (April 2021)	24.12 ± 2.78		23.59 ± 3.52		0.334
	0.044 *		0.248		
BMI difference	0.344 ± 1.18		0.194 ± 1.347		0.027 ^#^

* Statistically significant differences between the 1st and 2nd study. ^#^ Statistically significant differences between the groups.

**Table 3:** Average time spent on PA and meetings away from home.

		Group I Average ± SD			Group II Average ±SD		
		Before March 2020	March 2020–March 2021	After March 2021	Before March 2020	March 2020–March 2021	After March 2021
PA from Mon to Fri	min/per day	126 ± 102	86.6 ± 108	96 ± 114	84 ± 72	78 ± 90	84 ± 102
	1 vs2 vs3	0.000			0.080		
	GrIvs Gr II	0.001			0.001		
	GrIvs Gr II		0.767			0.767	
	GrIvs Gr II			0.777			0.777
PA per weekend	Min/per weekend	114 ± 90	66 ± 78	72 ± 72	84 ± 60	72 ± 66	78 ± 60
	1 vs2 vs3	0.000			0.178		
	GrI s Gr II	0.037			0.037		
	GrIvs Gr II		0.393			0.393	
	GrIvs Gr II			0.527			0.527
Time away from home from Mon to Fri	min/per day	540 ± 642	210 ± 342	444 ± 594	258 ± 132	264 ± 276	258 ± 300
	1 vs2 vs3	0.505			0.109		
	GrI s Gr II	0.008			0.008		
	GrIvs Gr II		0.002			0.002	
	GrIvs Gr II			0.086			0.086
Time away from home per weekend	Min/per weekend	390 ± 252	210 ± 192	342 ± 270	264 ± 138	240 ± 168	288 ± 246
	1 vs2 vs3	0.000			0.035		
	GrI s Gr II	0.009			0.009		
	GrIvs Gr II		0.144			0.144	
	GrIvs Gr II			0.322			0.322
Screen time from Mon to Fri	min/per day	4.9 ± 7.1	7.5 ± 2.3	5.5 ± 2.7	6.1 ± 3.7	6.6 ± 3.8	6.1 ± 3.8
	1 vs2 vs3	0.000			0.001		
	GrI s Gr II	0.001		0.001		0.001	
	GrIvs Gr II		0.028			0.028	
	GrIvs Gr II			0.713			0.713
Screen time per weekend	Min/per weekend	5.6 ± 11.9	7.9 ± 12.2	6.5 ± 12.3	5.0 ± 3.2	5.5 ± 3.6	4.8 ± 3.2
	1 vs2 vs3	0.000			0.008		
	GrI s Gr II	0.295			0.295		
	GrIvs Gr II		0.033			0.033	
	GrIvs Gr II			0.646			0.646

## Data Availability

The research results presented are part of a large ongoing study which has not yet been completed. If you are interested in specific data, please contact the first author—Agnieszka Chwałczyńska (agnieszka.chwalczynska@awf.woc.pl).
